# Dual effects of constitutively active androgen receptor and full-length androgen receptor for N-cadherin regulation in prostate cancer

**DOI:** 10.18632/oncotarget.18270

**Published:** 2017-05-29

**Authors:** Félicie Cottard, Pauline Ould Madi-Berthélémy, Eva Erdmann, Frédérique Schaff-Wendling, Céline Keime, Tao Ye, Jean-Emmanuel Kurtz, Jocelyn Céraline

**Affiliations:** ^1^ Université de Strasbourg, INSERM, FMTS, Strasbourg, France; ^2^ Université de Strasbourg, CNRS, INSERM, Institut de Génétique et de Biologie Moléculaire et Cellulaire, Illkirch-Graffenstaden, France; ^3^ Service d'Onco-Hématologie, Hôpitaux Universitaires de Strasbourg, Strasbourg, France

**Keywords:** androgen receptor, constitutively active androgen receptor variants, N-cadherin, EMT, prostate cancer

## Abstract

Constitutively active androgen receptor (AR) variants have been involved in the expression of mesenchymal markers such as N-cadherin in prostate cancer (PCa). However, the underlying molecular mechanisms remain elusive. It remains unclear, whether N-cadherin gene (CDH2) is a direct transcriptional target of AR variants or whether the observed upregulation is due to indirect effects through additional regulatory factors. Moreover, the specific contribution of full-length AR and AR variants in N-cadherin regulation in PCa has never been explored deeply. To investigate this, we artificially mimicked the co-expression of AR variants together with a full-length AR and performed miRNA-seq, RNA-seq and ChIP assays. Our results were in favor of a direct AR variants action on CDH2. Our data also revealed a distinctive mode of action between full-length AR and AR variants to regulate N-cadherin expression. Both wild type AR and AR variants could interact with a regulatory element in intron 1 of CDH2. However, a higher histone H4 acetylation in this genomic region was only observed with AR variants. This suggests that full-length AR may play an occluding function to impede CDH2 upregulation. Our data further highlighted a negative effect of AR variants on the expression of the endogenous full-length AR in LNCaP. These differences in the mode of action of AR variants and full-length AR for the control of one key gene for prostate cancer progression could be worth considering for targeting AR variants in PCa.

## INTRODUCTION

The androgen receptor (AR), a member of the nuclear receptor superfamily, is a ligand-dependent transcription factor that controls the development and the normal function of the prostate gland [1]. The AR is also involved in the development of prostate cancer (PCa) and current treatments for metastatic disease are based on the inhibition of androgen signaling pathways [2–4]. Unfortunately, this therapy is highly, but only transiently effective, as most patients relapse after approximately 1.5 years of treatment and progress toward castration resistant PCa (CRPC). To date, it is clearly accepted that the reactivation of the androgen/androgen receptor-signaling axis is a major event in the onset of CRPC. The mechanisms of this reactivation are numerous and include molecular events such as AR amplification, AR mutations, overexpression of AR cofactors, increased intratumoral androgen synthesis, ligand-independent AR activation by cytokines or growth factors, and constitutively active AR variants [5–9].

Constitutively active AR variants are due to nonsense mutations in exon 4 (e.g. AR-Q640X) [6, 10–12] or alternative splicing of the AR mRNA (e.g. AR-V7) [12–19] resulting in premature termination of the AR protein. These AR variants are defective in ligand binding and display ligand-independent transcriptional activities in PCa cells [10, 11, 13]. AR variants support PCa cells growth both *in vitro* and *in vivo* in an androgen-depleted environment and lead to resistance to novel therapies such as enzalutamide and abiraterone acetate [14, 20–23]. Hence, there is an urgent need to understand the mode of action of constitutively active AR variants in order to find novel therapeutic targets in CRPC.

In addition to their role in castration resistance, a number of studies suggest that constitutively active AR variants are involved in PCa progression. In the first place, AR^v567es^ induces autonomously prostate tumorigenesis and furthermore leads to invasive adenocarcinoma after castration [21]. Moreover, our previous data show that AR variants are associated with a partial epithelial mesenchymal transition (EMT) as evidenced by the co-expression of epithelial and mesenchymal markers in PCa cells. Indeed, AR-Q640X and AR-V7 lead to an upregulation of N-cadherin, vimentin and ZEB1 in LNCaP cells without decreasing the expression of E-cadherin [24]. Besides, a link between AR-V7 and ZEB1 upregulation has been reported in 22RV1 and LNCaP cells [25]. An increased expression of N-cadherin, vimentin, Snail and Twist is observed in an AR-V7 transgenic mouse model [26]. EMT promotes a modification of cell shape favoring tumor migration and invasion and increasing evidences have demonstrated a role of AR variants in PCa cells migration [25, 27–29]. All together, these data show that constitutively active AR variants induce a particular set of genes contributing to tumor progression during CRPC. This was reinforced by several studies showing that the full-length AR (AR-FL) and AR variants induce distinct transcriptional programs [28, 30]. However, the mechanisms leading to this differential expression of genes are currently unknown and need to be elucidated in order to have a better understanding towards the role of AR variants in tumor progression.

N-cadherin is involved in cell adhesion and promotes tumor progression owing to its role in cell migration, invasion and survival [31]. As mentioned above, we have shown that constitutively active AR variants induce N-cadherin expression [24]. Interestingly, AR-FL and AR variants compete against each other to regulate N-cadherin expression [24]. In the present study, we explored the mechanisms leading to N-cadherin expression in the presence of constitutively active AR variants. We hypothesized that at least four mechanisms may be involved in N-cadherin upregulation in the presence of AR variants. In the first place, AR variants may induce the expression of a transcription factor known to regulate N-cadherin expression. Secondly, as AR-FL induces or represses miRNAs, AR variants may differentially modulate these miRNAs, potentially resulting in N-cadherin upregulation. Thirdly, as 13 androgen response elements (AREs) have been described in intron 1 of the N-cadherin *CDH2* gene [32], we postulated that AR variants may directly regulate N-cadherin expression by interacting with these AREs. Finally, as we have shown that AR-FL and AR variants compete against each other to regulate N-cadherin expression, we hypothesized that AR variants may downregulate AR-FL in PCa cells. In this work, we have deciphered the mechanisms of AR variants-induced N-cadherin differential expression, showing that N-cadherin upregulation in prostate cancer cells appears to result from the binding of AR variants to AREs in intron 1 of the *CDH2* gene followed by histone H4 acetylation, but also from a decrease of endogenous AR-FL. These data emphasize the role of AR variants in the progression of CRPC.

## RESULTS

### Analysis of the impact of AR variants on potential N-cadherin regulators

**Figure 1:**
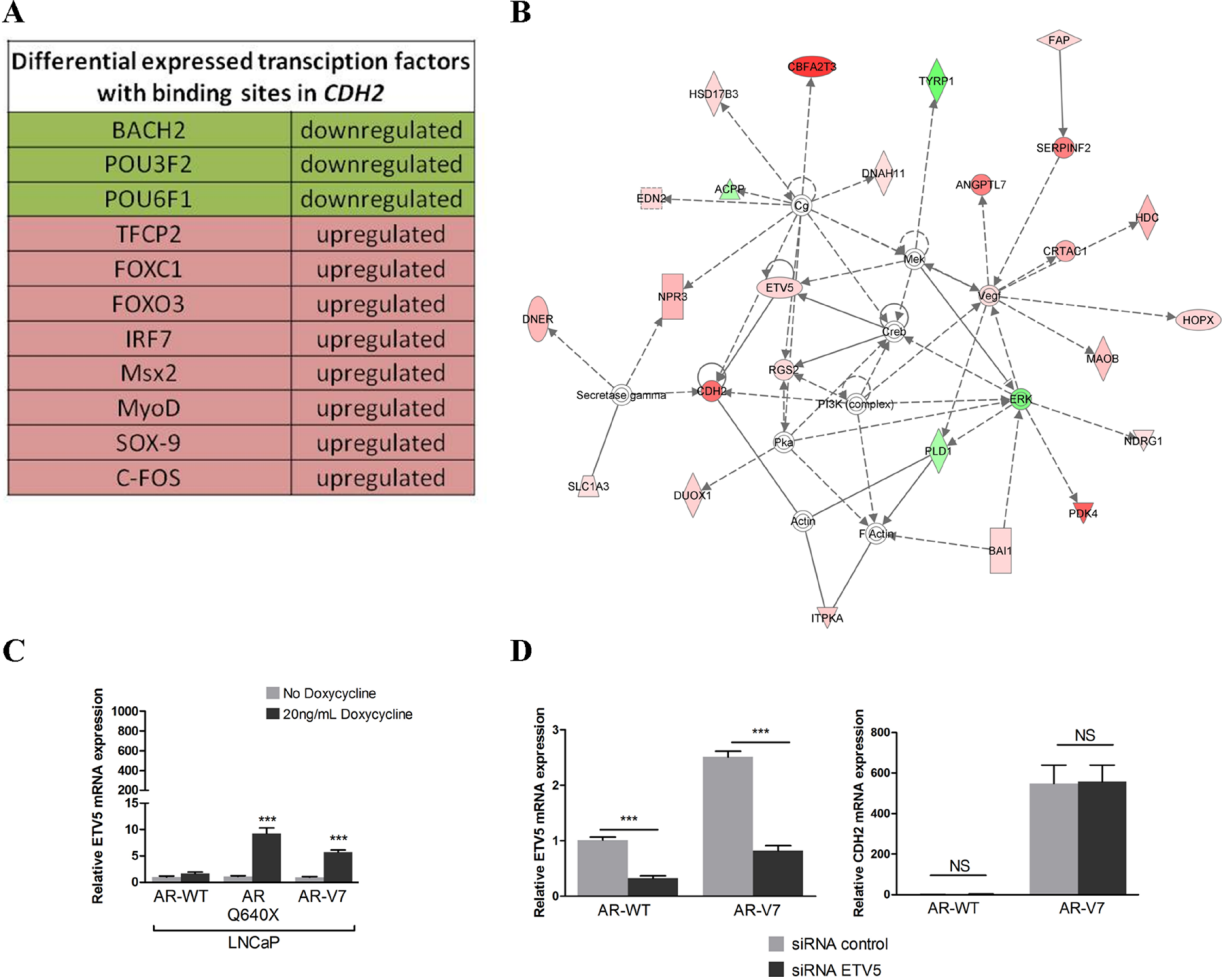
ETV5 and SOX9 do not regulate N-cadherin (**A**) Transcriptional regulators deregulated in the presence of AR-V7 compared to AR-WT with known binding sites in *CDH2* gene are listed. (**B**) IPA analysis from deregulated genes in the presence of AR-V7 highlighted a network in which ETV5 was a direct regulator of CDH2. Green: downregulated expression; Red: upregulated expression. (**C**) A lentiviral inducible system was used to verify ETV5 expression in the presence of AR variants observed in our RNA-seq data. AR-WT and AR variants expression were induced with 20 ng/mL doxycycline. LNCaP expressing AR-WT and AR variants were cultured in the presence of 10nM DHT and ETV5 mRNA expression level was analyzed by real-time PCR 4 days after induction. (**D**) To analyze the impact of ETV5 on N-cadherin expression, AR-WT and AR-V7 were induced in LNCaP with 20 ng/mL doxycycline and cells were transfected with 50 nM of siRNA against ETV5 (left panel). After 48 h, total mRNA was extracted and *CDH2* mRNA expression level was assessed by qRT-PCR (right panel). For all qRT-PCR analyses, the results were normalized to β-ACTIN. Relative expression is represented as the mean of ΔΔCt ± SEM of three independent experiments. NS: not significant, ***P < 0.001, two-tailed Student's *t*-test.

We have previously shown a difference in the capacity of full-length AR (AR-FL) and AR variants to upregulate N-cadherin expression in PCa cells [24]. We investigated whether AR variants could directly deregulate known N-cadherin transcriptional regulators. We performed an RNA-seq experiment in LNCaP cells overexpressing AR-WT or AR-V7. From this analysis, we identified 751 upregulated genes and 108 downregulated genes in LNCaP cells overexpressing AR-V7 compared to AR-WT (Filter criteria: log2Fold-Change > 1 and an adjusted *p*-value for multiple testing < 0.05). First, biological functions of deregulated genes were studied using IPA software. Interestingly, deregulated genes in the presence of AR-V7 in LNCaP cells were associated with functions such as cellular movement, cell morphology or cellular assembly and organization (Supplementary Figure 1). As expected, CDH2 was found in the list of upregulated genes involved in these functions. In the next step, we searched, using two different methods, the differentially expressed genes that encode for transcription factors able to regulate N-cadherin expression. First, we identified differentially expressed transcription factors in the presence of AR-V7 with predicted binding sites in *CDH2* gene using SABiosciences proprietary database DECODE (DECipherment Of DNA Elements) (Figure 1A). Among these factors, only SOX9 has been experimentally linked to N-cadherin expression [33]. We further looked to confirm the increased expression of SOX9 in the presence of AR-V7 compared to AR-WT in our LNCaP model. We used a doxycycline inducible system for the expression of AR variants in LNCaP cells followed by qRT-PCR and Western Blot analyses. Unfortunately, we did not observe any increase in SOX9 expression in the presence of AR-V7 (data not shown). We continued the RNA-seq analysis using the IPA software and identified a network in which ETV5 was a direct regulator of N-cadherin in our model (Figure 1B). The increase of ETV5 mRNA levels in the presence of AR-V7 was confirmed with the doxycycline inducible AR-V7 expression system (Supplementary Figure 2, Figure 1C). Nevertheless, ETV5 protein was not detected in immunoblots from LNCaP cells overexpressing AR variants (Supplementary Figure 3). Finally, we investigated whether ETV5 silencing could decrease N-cadherin upregulation in the presence of AR variants. We used the doxycycline inducible AR-V7 expression system and ETV5 specific siRNAs (Figure 1D, left panel) and could not observe any change in N-cadherin expression in the presence of AR-V7 (Figure 1D, right panel). Taken together, our data exclude two main potential transcription factors that could indirectly lead to N-cadherin expression in the presence of AR variants.

### MiR-221–3p and miR-26b-5p are not involved in N-cadherin upregulation in the presence of AR variants

**Figure 2:**
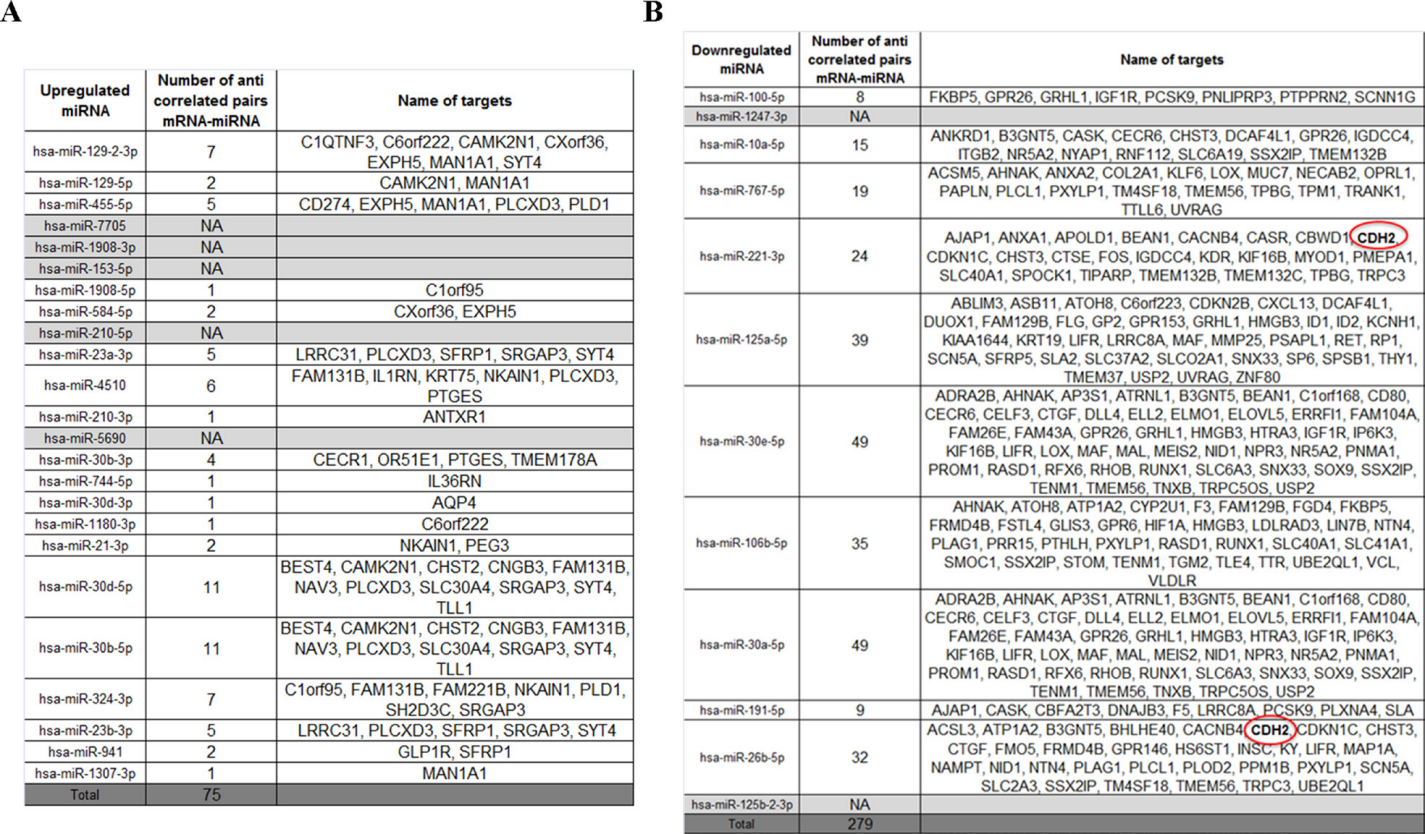
Integrative analysis between RNA-seq and miRNA-seq An integrative analysis was performed using “micro RNA target filter” in IPA software to identify potential targets of microRNAs. Targets of upregulated (**A**) and downregulated (**B**) microRNAs are listed in the following table.

MicroRNA deregulation could be another mechanism that indirectly links AR variants to N-cadherin expression. Indeed, microRNAs targeting N-cadherin mRNA could be decreased in the presence of AR variants and, in turn, could explain the upregulation of N-cadherin in our model. Hence, from the same samples used for RNA-seq, we performed a miRNA-seq analysis in order to identify deregulated microRNAs in the presence of AR variants. This analysis highlighted 12 downregulated microRNAs and 24 upregulated microRNAs in the presence of AR-V7 compared to AR-WT with an adjusted *p*-value ≤ 0.05 (Figure 2).

First, we checked if microRNAs known to directly regulate N-cadherin were modulated by the presence of AR variants. To date, few microRNAs are described as direct regulators of N-cadherin expression. Indeed, miR-145 is able to modulate N-cadherin expression in the prostate cancer cell line PC-3 [34]. Moreover, miR-218 is known to directly regulate N-cadherin expression in aggressive lung adenocarcinoma [35]. miR-369–3p, miR-496 and miR-543 are also direct regulators of N-cadherin during neurogenesis and neuronal migration [36]. However, according to our miRNA-seq, the expression levels of miR-145, miR-218, miR-369–3p, miR-496 and miR-543 were not affected in our model.

To go further in our analysis, we performed an integrative analysis between the RNA-seq and miRNA-seq using “micro RNA target filter” in IPA software to search for potential targets of microRNAs and we obtained a list of mRNA-microRNA pairs. Because microRNAs negatively regulate the expression of mRNA, we have selected only pairs with an anticorrelated expression pattern. Finally, we have obtained 75 potential pairs for upregulated microRNAs and 279 pairs for downregulated microRNAs (Figure 2).

In particular, the miRNA-seq analysis revealed that miR-221–3p and miR-26b-5p could regulate N-cadherin expression. However, according to our miRNA-seq data, the expression of miR-221–3p and miR-26b-5p is only decreased by a 1.2-fold and 1.1-fold respectively in the presence of AR-V7 compared to AR-WT. Moreover, this slight decrease was not confirmed by qRT-PCR (data not shown). Taken together, these results reveal that neither miR-221–3p nor miR-26b-5p is involved in N-cadherin upregulation in our model.

### AR variants bind androgen response elements in intron 1 of CDH2 to upregulate N-cadherin expression

**Figure 3:**
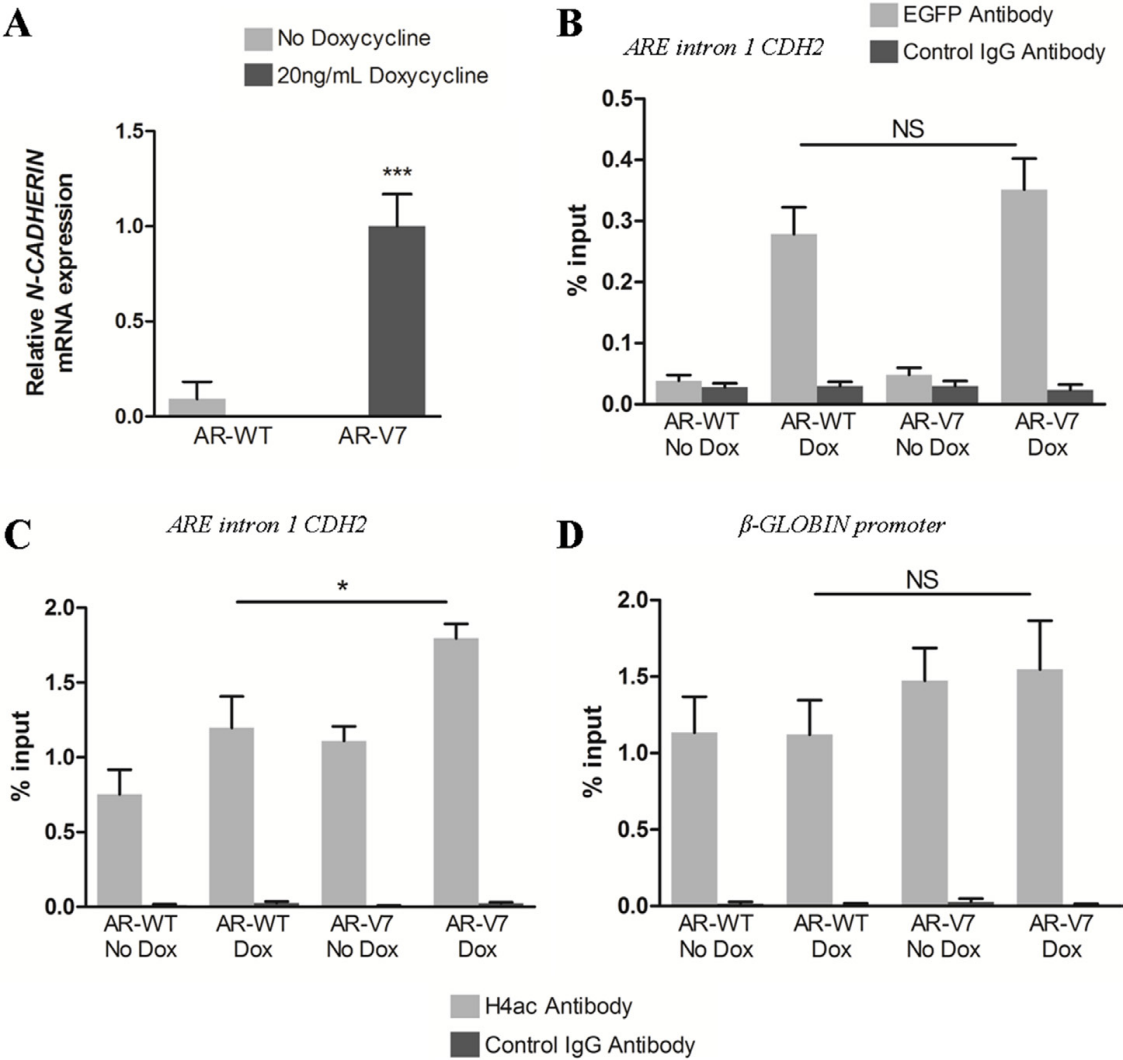
Both AR-FL and AR variants are recruited at ARE in *CDH2* intron 1 but AR variants are associated with increased histone H4 acetylation EGFP-tagged AR-WT and AR-V7 expression were induced with 20 ng/mL doxycycline in LNCaP cells and cells were cultured in complete medium supplemented with 10 nM DHT. (**A**) Twenty-four hours after induction, N-cadherin expression was analyzed by qRT-PCR. Recruitment of AR-WT and AR-V7 (**B**) and histone H4 acetylation level (**C**) at ARE in *CDH2* intron 1 were analyzed by ChiP-qPCR using respectively anti-EGFP antibody or anti-Acetyl H4 (H4ac) antibody. (**D**) Histone H4 acetylation level at β-GLOBIN promoter was examined as control. Control IgG antibody was used to determine the specificity of the reaction. The enrichment of EGFP-AR and histone H4 acetylation was calculated using a standard curve with serial dilutions of the input for each primer. Results were represented as the mean of %input ± SEM of three independent experiments. ***P < 0.001, *P < 0.05, NS: Not significant, two-tailed Student's *t*-test.

It has been shown that AR-FL can be recruited to the 13 ARE repeats present in intron 1 of *CDH2* [32]. To highlight a potential difference between the recruitment of AR-FL and AR variants to these AR binding sites, ChIP-qPCR experiments were conducted in LNCaP cells 24 h after doxycycline-induced expression of the full-length AR-WT (EGFP-AR-WT) or the AR-V7 variant (EGFP-AR-V7) (Figure 3; Supplementary Figure 4). As expected, N-cadherin upregulation was only observed in the presence of AR-V7 (Figure 3A). Surprisingly, there was no significant difference between AR-WT and AR-V7 recruitment to the AREs present in intron 1 of *CDH2* (Figure 3B). Hence, it seems that additional mechanisms are required to explain why only AR variants are associated with N-cadherin upregulation. To go further, ChIP-qPCR experiments were conducted to analyze histone H4 acetylation level, a mark of active chromatin in the region encompassing the AREs in intron 1 of *CDH2*. The level of histone H4 acetylation in LNCaP cells expressing AR-WT was comparable to that one obtained in non-induced control cells (Figure 3C). Moreover, AR-V7 led to a significant increase of histone H4 acetylation at AR binding sites in *CDH2* intron 1 (Figure 3C), but not at the control β-GLOBIN promoter (Figure 3D). Similar results were obtained 72 h after AR-V7 or AR-Q640X induction in LNCaP cells (Supplementary Figure 5) and also in the prostate cancer C4–2B cells (Supplementary Figure 6). Furthermore, we highlighted the necessity of a functional AR DNA binding domain (DBD) for N-cadherin upregulation using a mutant AR-V7 with the C576Y mutation in the first Zinc finger (Supplementary Figure 7). In conclusion, our data indicate that both AR-FL and AR variants can be recruited to the AREs present in intron 1 of *CDH2*, but only AR variants lead to an increase of histone H4 acetylation.

### The loss of AR-FL in the presence of AR variants increases N-cadherin upregulation

**Figure 4:**
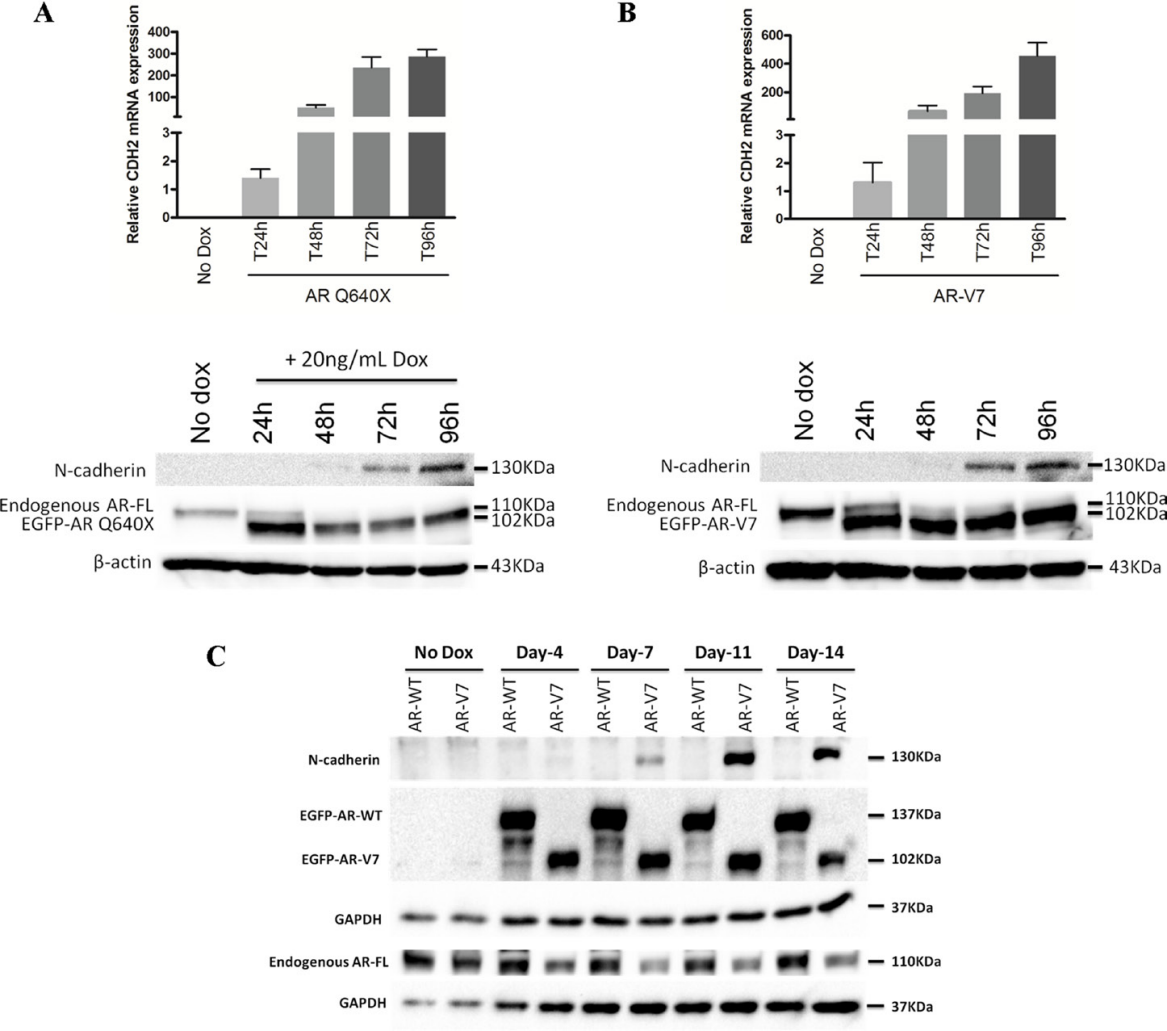
The expression of AR variants is associated with a loss of endogenous AR-FL EGFP-tagged AR-WT, AR-Q640X and AR-V7 expression were induced with 20 ng/mL doxycycline in LNCaP and cells were cultured in complete medium supplemented with 10 nM DHT. (**A**) (**B**) Short-term effects of AR variants (from 24 h to 96 h after induction) on N-cadherin and AR-FL expression by qRT-PCR (upper panel) and Western Blot (lower panel). Results show a gradual decrease of AR-FL upon induction of AR variants. (**C**) Long-term effects of AR variants (from 4 days to 14 days) on N-cadherin and AR-FL expression by Western Blot. Endogenous AR-FL was detected using a specific antibody targeting the C-terminal extremity of AR (AR-C19 antibody). β-actin and GAPDH were used as a loading control. These experiments were repeated at least three times. The images from Western Blot were cropped to remove the parts which contain no information.

The above mentioned results indicating that AR-FL binds AREs in intron 1 of *CDH2* but without effect on N-cadherin expression coupled with our previous observation that DHT-activated AR-FL antagonizes the ability of AR variants to activate N-cadherin gene [24], suggests that DHT-activated AR-FL may occlude these AR binding sites in intron 1 of *CDH2* and prevent AR variant binding. This model argues that the AR variants must have additional properties in order to overcome the occluding effects of DHT-activated AR-FL and to enhance N-cadherin expression. To explore this issue, we evaluated by Western Blot, the expression kinetics of N-cadherin and endogenous AR-FL in LNCaP cells in the presence of DHT-activated AR-WT or AR variants (Figure 4). Interestingly, N-cadherin upregulation by AR variants was concomitant with a loss of endogenous AR-FL expression (Figure 4A, 4B). Moreover, the decrease of endogenous AR expression was only observed in LNCaP overexpressing AR-V7 but not in the presence of DHT-activated AR-WT (Figure 4C, Supplementary Figure 8). This downregulation was also observed when C4–2B cells overexpressed AR-Q640X variant (Supplementary Figure 6). These data indicate that AR variants downregulate AR-FL to overcome its occluding effects on AR binding sites in intron 1 of *CDH2*.

## DISCUSSION

To date, it is accepted that constitutively active androgen receptor variants play a key role in castration resistance owing to their exclusively nuclear localization and their constitutive transcriptional activity in the absence of androgens [10–14, 16–19]. Moreover, during these last years, several data suggested that AR variants could also promote tumor progression. Indeed, AR variants were associated with an expression of EMT markers such as N-cadherin, vimentin or SNAIL in *in vitro* and *in vivo* models [21, 24, 25]. Furthermore, RNA-seq data reveal that AR variants regulate a subset of genes preferentially involved in cell cycle [15, 30]. However, the mechanisms associated with this distinct expression profile are poorly studied. Hence, in the present study, we have explored the mechanisms by which AR variants induce this distinct subset of genes through focusing our research on N-cadherin expression.

Our data from ChiP-qPCR evidenced that both AR-FL and AR variants were able to bind to the AREs present in intron 1 of the *CDH2* gene. In accordance with our findings, the analysis of previously reported ChiP-seq data confirms AR-FL binding to an ARE in intron 1 of *CDH2* [37]. Our observation corroborates the ChiP-seq analysis performed in 22rv1 CRPC cells showing an enrichment of AR variants in this region [38]. Furthermore, another ChiP-seq analysis supports the concept that the genome-wide binding preference of AR variant AR^v567es^ is identical to androgen-activated AR-FL [39]. However, our ChiP experiment data highlighted a remarkable difference between AR-FL and AR variants following their binding to AREs in two different cell lines. Indeed, AR variant binding to AREs in intron 1 of the *CDH2* gene was associated with an increase in histone H4 acetylation, a positive marker of gene activation. These data provide a novel stage of knowledge regarding the mode of action of AR variants in prostate cancer cells. Both AR-FL and AR variants bind to the same regulatory region, but the signal triggered is different. In the context of *CDH2* gene, AR variant binding is a positive signal for gene transcription, while this is not the case for DHT-activated AR-FL. Indeed, AR variants could be involved in a transcriptional activation complex by the recruitment of histone acetyltransferase (HAT) to induce N-cadherin expression. Besides, AR-FL could occlude the AR binding sites and prevent AR variant activity. Since the ligand-binding domain (LBD) is an important platform for the interaction of cofactors, the loss of this region in AR variants could partly explain the distinct transcriptional programs in the presence of AR-FL or AR variants [40]. Consistent with this hypothesis, the recruitment of the co-activator GRIP-1 was impaired in the presence of AR-Q640X [41].

**Figure 5:**
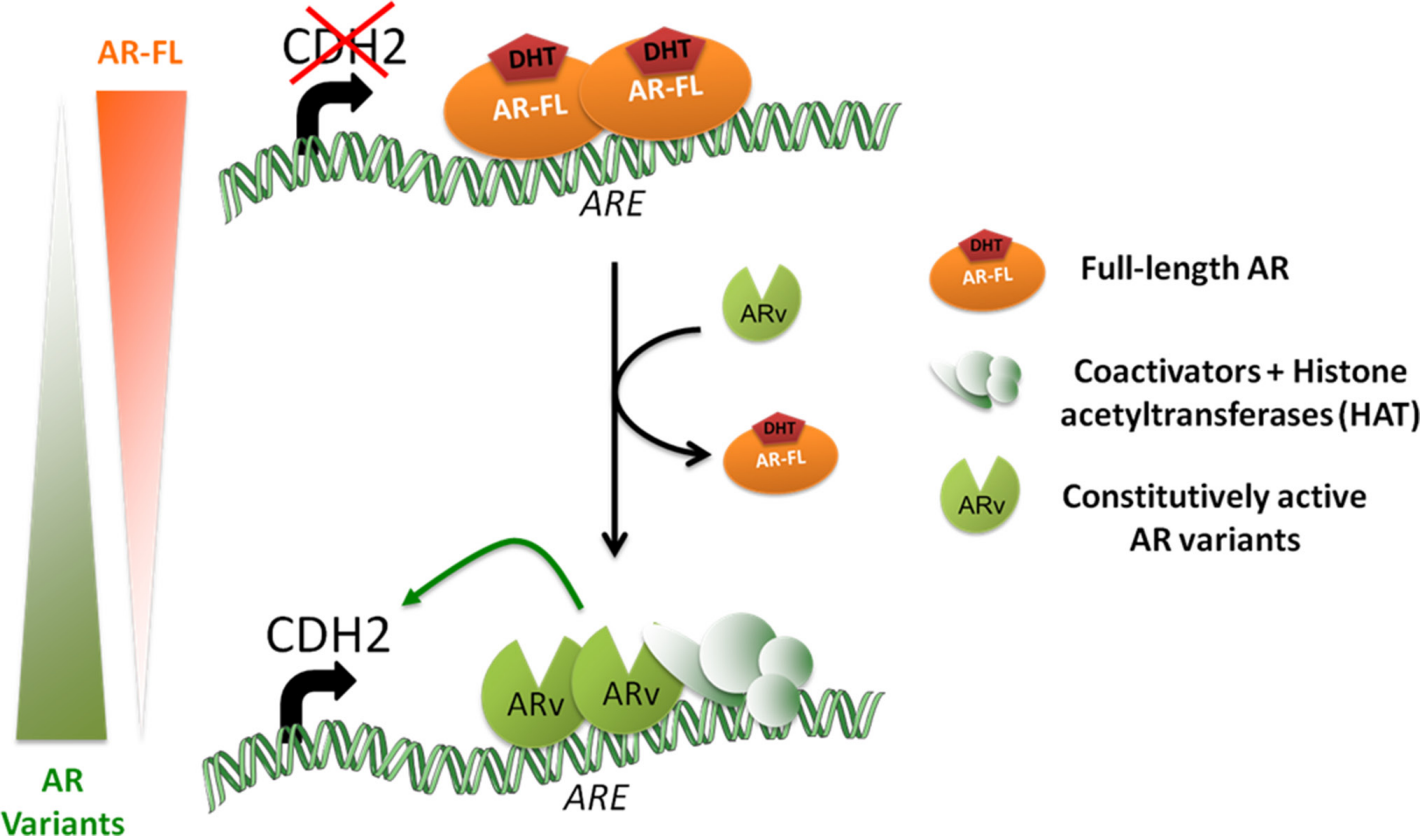
Proposed model of N-cadherin regulation by androgen receptor in LNCaP cells AR-FL and AR variants differ in their mode of action to regulate N-cadherin. AR-FL binds AREs in intron 1 of *CDH2* but is unable to induce N-cadherin expression. In contrast, AR variants binds also AREs and promote N-cadherin transcription by the recruitment of a transcriptional activation complex with histone acetyltransferase (HAT) activity as evidenced by the more important histone H4 acetylation of this region in the presence of AR variants compared to AR-FL. Moreover, the gradual loss of AR-FL observed when AR variants are expressed may potentiate their effects on N-cadherin expression.

In our model, as observed in human CRPC samples, we have a co-expression of AR-FL and AR variants. As suggested in our previous work, AR-FL and AR variants could compete each other to regulate N-cadherin expression [24]. Here, we have shown that AR variants were associated with a decrease of endogenous AR-FL in LNCaP cells and C4–2B cells. A similar decrease was also observed in the absence of DHT. This AR-FL downregulation was also reported in the presence of a different truncated AR variant [28]. Moreover, the AR-FL loss was concomitant with the upregulation of N-cadherin. Taken together, our results and recently published data led us to propose the following model (Figure 5) [42–44]. The loss of AR-FL may attenuate competitive DNA binding between AR-FL and AR variants, and potentiate AR variants activities on *CDH2* gene. Further analyses need to be performed to validate this model and to understand how AR variants induce a decrease of endogenous AR-FL in prostate cancer cells. In the present study, we focused only on N-cadherin expression, but, it may be interested to study if the increased expression of other mesenchymal markers observed in the presence AR variants occurs following the same mechanism. Indeed, in our previous study, we showed an increased expression of vimentin, ZEB1 and SNAIL [24]. Interestingly, Miao and colleagues showed recently that AR-FL is a negative regulator of SNAIL expression by interacting with AREs in the promoter region. Conversely, AR variants are unable to bind this locus [45]. These data combined with our results suggest that the upregulation of SNAIL in the presence of AR variants in our model could result from the loss of AR-FL induced by AR variants. This could be another mechanism whereby AR variants induce mesenchymal markers expression, but, this hypothesis remains to be studied.

Using RNA-seq analysis, we identified ETV5 and SOX9 as potential regulators of N-cadherin. Indeed, previous studies have reported a link between N-cadherin and ETV5 or SOX9 expression. For example, the overexpression of ETV5 in endometrial cancer cells was associated with an increased expression of N-cadherin [46]. Moreover, SOX9 could regulate N-cadherin by interacting with *CDH2* promoter [33] There are also two other SOX9 motifs in the intron 1 of *CDH2*. Here, in our model the increased expression of ETV5 revealed in the RNA-seq was confirmed by qRT-PCR, but at the protein level, ETV5 expression was not detectable in immunoblots. Moreover, ETV5 downregulation by siRNA was not associated with N-cadherin downregulation, suggesting that ETV5 could not be the leading transcription factor induced by AR variants to upregulate N-cadherin expression in our model. The upregulation of SOX9 expression observed in our RNA-seq data was not confirmed at mRNA and protein levels. Anyway, the association between ETV5 or SOX9 expression level and the presence of AR variants in prostate cancer cells is not clear. In a first study, a DNA microarray analysis performed in LNCaP cells overexpressing AR-V7 revealed an upregulation of ETV5, but not SOX9 [30]. Conversely, ETV5 and SOX9 were not affected by AR-V7 knockdown in CWR-R1 and 22Rv1 cancer cells [14]. Likewise, the overexpression of a truncated AR variant in LNCaP cells was not associated with an overexpression of these transcription factors [28]. However, we cannot exclude the possibility that another transcription factor not studied here could lead to N-cadherin expression in our model.

Since Epithelial Mesenchymal Transition (EMT) was generally associated with a particular miRNA expression profile, we wondered whether N-cadherin induced by AR variants could be also regulated by microRNAs. So, using an integrative analysis between miRNA-seq and RNA-seq, we have examined microRNAs with a decreased expression in the presence of AR-V7 compared to AR-WT and selected the ones that were known to target the 3′UTR extremity of *CDH2*. From this analysis, we have identified two microRNAs, miR-221–3p and miR-26b-5p, able to target 3′UTR extremity of *CDH2* with a high score of prediction. However, the decreased expression of miR-221–3p and miR-26b-5p observed in the miRNA-seq analysis was not confirmed by qRT-PCR. The link between these two microRNAs and N-cadherin was only based on bioinformatics prediction. Indeed, the direct regulation of N-cadherin by miR-221–3p or miR-26b-5p was never described in previous reports. Nevertheless, in hepatocellular cancer cells, miR-26b-5p regulates Epithelial Mesenchymal Transition. Indeed, its overexpression is associated with an increase of E-cadherin expression and a decrease of vimentin expression. However, the authors have not analyzed N-cadherin expression in the presence of miR-26b-5p [47]. In contrast, miR-221–3p is rather overexpressed in cancer and its expression is associated with a more proliferative and invasive phenotype [48, 49]. In the presence of AR variants, only miR-100–5p and miR-1247–3p were downregulated with a fold change of at least 1.5. However, their impact on N-cadherin expression is unknown. In conclusion, our findings are not in favor of N-cadherin regulation by microRNAs in our model.

In summary, in this study we bring evidence that N-cadherin upregulation in prostate cancer cells appears to result from the binding of AR variants to AREs in intron 1 of the *CDH2* gene followed by histone H4 acetylation, but also from a decrease of endogenous AR-FL. These data emphasize the role of AR variants in the progression of CRPC and highlight the importance to develop drugs targeting these variants or their mode of action.

## MATERIALS AND METHODS

### Cell culture

LNCaP cells, clone FGC (ECACC) and C4–2B cell line (ViroMed Laboratories, Minnetonka, MN, USA) were maintained in RPMI-1640 media supplemented with 10% fetal bovine serum (FBS), 10 mM HEPES, 2 mM L-glutamine, 100 U/mL penicillin, 100 μg/mL streptomycin (Sigma-Aldrich) and 1 mM pyruvate (Invitrogen) (complete medium). HEK 293T cells were grown in Dulbecco's modified Eagle's medium (DMEM) containing 10% FBS, 2 mM L-glutamine, 100 U/mL penicillin and 100 μg/mL streptomycin (Sigma-Aldrich).

### Plasmids

For lentiviral infection, the full-length wild type androgen receptor (AR-WT) and the constitutively active AR variants, AR-Q640X and AR-V7, were excised from previously described pEGFP-AR [11, 12] using XhoI/BamHI and cloned in pENTR4-GFP-C3 (Addgene) between XhoI and BamHI restriction sites. Then, pENTR4-GFP-AR was recombined with pLenti PGK Blast DEST (Addgene) using Gateway LR clonase II enzyme mix (Life Technologies). For lentiviral inducible expression, the Lenti-X^TM^ Tet-ON^®^ 3G Inducible Expression System (Clontech) was used. Briefly, cDNA of EGFP-AR-WT, EGFP-AR-Q640X and EGFP-AR-V7 were amplified from previously described pEGFP-AR using specific primers (Supplementary Table 1). Each cDNA amplicon was cloned in pLVX-TRE3G vector between BamHI/MluI using the In fusion^®^ HD Cloning Kit (Clontech).

### Lentiviral transduction

Lentivirus expressing GFP, AR-WT, AR-Q640X and AR-V7 were prepared by co-transfecting 6 × 10^6^ HEK 293T with 9 μg of packaging plasmids (pLP1, pLP2, and pLP/VSVG, ratio 1:1:1) and 3 μg of pLenti-PGK-AR or 1 μg pLenti-PGK-GFP as control using respectively 36 μl or 30 μl of lipofectamine 2000 (Invitrogen). Cells were transfected in antibiotic-free medium (DMEM containing 10% FBS and 2 mM L-glutamine). Culture media were changed the day after and 72 h after transfection, culture media were recovered, centrifuged at 3000 rpm for 15 min to pellet debris. The viral supernatants were filtered through a Millex-HV 0.45 μm, concentrated 10x with Amicon^®^ Ultra-15 Centrifugal Filters Ultracell^®^-100K (Millipore). Then, LNCaP cells were incubated with 1:10 of concentrated viral supernatant in LNCaP complete medium supplemented with 10 nM DHT and 6 μg/mL of polybrene (Sigma Aldrich) for maximal transduction efficiency. These conditions lead to almost 100% of transduced cells. For miRNA-seq and RNA-seq experiments, a pool of transduced cells was used and the transduction efficiency was determined by analyzing EGFP level by fluorescent microscopy and qRT-PCR.

### Lentiviral inducible system

To establish Tet-3G-expressing stable LNCaP and C4–2B clones, Tet-3G lentiviral particles produced in HEK 293T cells were used to transduce LNCaP and C4–2B cells. Stable clones were selected with 400 μg/mL geneticin (Life Technologies). For an inducible expression of AR variants, Tet-3G stable LNCaP and C4–2B cells were transduced with pLVX-TRE3G-EGFP-AR-WT, pLVX-TRE3G-EGFP-AR-Q640X and pLVX-TRE3G-EGFP-AR-V7 lentiviral particles, and transduced LNCaP and C4–2B cells were selected with 300 μg/mL geneticin and 400 ng/mL or 100 ng/mL puromycin (Life Technologies) respectively. AR expression was induced using 20 ng/mL doxycycline (Life Technologies).

### siRNA transfection

Two days before transfection, AR doxycycline-inducible LNCaP cells were plated into 12-wells plates. Prior to transfection, medium was refreshed with RPMI-1640 supplemented with 10% Tet System Approved FBS (catalog no. 8630–1, BD biosciences), 10 mM HEPES, 2 mM L-glutamine, 100 U/mL penicillin, 100 μg/mL streptomycin (Sigma-Aldrich), 1 mM pyruvate (Invitrogen), 10 nM DHT and 20 ng/mL doxycycline (Life technologies). Cells were transfected with 50 nM siRNA against ETV5 (Hs_ETV5_5, catalog no. SI03019394, Qiagen) using 3 μL of JET-PRIME (Polyplus Transfection, Ozyme). AllStars Negative Control siRNA (catalog no. 1027280, Qiagen) was used as control. After 48 h, total RNA was extracted and ETV5 and CDH2 expression levels were analyzed by qRT-PCR.

### Quantitative real-time PCR

Total RNA was isolated using NucleoSpin^®^ RNA II assay (Macherey-Nagel) according to the manufacturer's procedure and 400 ng of total RNA were reverse transcribed using iScript kit (Bio-Rad). Real-time PCR was conducted using GoTaq^®^ qPCR Master Mix (Promega) and validated primers (Supplementary Table 2, QuantiTect Primers, Qiagen). *PBGD* and *B-ACTIN* mRNA expression were used as internal control for normalization. The relative expression of target gene was determined by the ΔΔ Ct method.

### Western blot analysis

Transduced cells were lysed in RIPA buffer (Pierce, Thermo Scientific) supplemented with 1x phosphatase inhibitor, 1x protease inhibitor (Sigma Aldrich) and 250 U/mL Benzonase (Millipore). Protein concentration was quantified using BCA Protein Assay (Pierce Biotechnology) according to the manufacturer's protocol. An equivalent quantity of total proteins was separated by 7.5% SDS-PAGE or by TGX Stain-Free^TM^ FastCast^TM^ Acrylamide gel, 12% (cat. #161–0184) and transferred onto a nitrocellulose membrane. Membranes were blocked with PBS/0.1% Tween/4%nonfat dry milk and probed with primary antibodies against EGFP (1:200, sc-9996, Santa Cruz Biotechnology), ETV5 (1:2000, catalog no. #MABN683, Millipore), AR (clone G122–434) (1:500, catalog no. 554225, BD Biosciences), AR-C19 (1:200, sc-815, Santa Cruz Biotechnology), N-cadherin (1:2500, catalog no. 610920, BD Biosciences), b-actin (1:2000, sc-47778, Santa Cruz Biotechnology) or GAPDH (1:1000, sc-20357, Santa Cruz Biotechnology) at 4°C overnight. Blots were washed and incubated with horseradish peroxidase-conjugated goat anti-mouse (1:2000, sc-2005, Santa Cruz Biotechnology), goat anti-rabbit (1:5000, sc-2004, Santa Cruz Biotechnology), rabbit anti-goat (1:2000, sc-2768, Santa Cruz Biotechnology) or rat anti-mouse IgG2a (1:1000, catalog no. 553391, BD Biosciences) secondary antibodies for 1 h. Immunoreactive proteins were visualized by chemiluminescence (Immobilon^TM^ Western, Millipore). For Stain-Free gels, total protein normalization was performed using Image Lab^TM^ Software (Bio-Rad Laboratories).

### Chromatin immunoprecipitation (ChiP)

To analyze the occupancy of AR in *CDH2* gene, we have performed a ChiP using ChiP-IT^®^ High Sensitivity (Active Motif) according to the manufacturer's protocol. Briefly, 24 h or 72h after doxycycline induction, LNCaP and C4–2B overexpressing AR-WT, AR-V7 or non-induced cells as control were crosslinked with 1.1% of paraformaldehyde and fixation buffer for 15 min at room temperature and the reaction was quenched with Stop Solution. After cell lysis, chromatin was sonicated 2.5 h using qSONICA Q800R (20sec ON/40sec OFF). Then, 30 μg of chromatin was immunoprecipitated with 5 μg of anti-EGFP antibody (kindly provided by Dr. K. WHITE, Institute for Genomics and Systems Biology, University of Chicago) or goat IgG isotype (sc-2028, Santa Cruz Biotechnology). Immunoprecipitated DNA was incubated with agarose beads for 3 h, extensively washed, eluated, reverse-crosslinked and purified according to the protocol. To analyze histone H4 acetylation in *CDH2* gene, 10 μg of chromatin was incubated overnight with 10 μg of Histone H4ac (pan-acetyl) antibody (n° 39925, Active Motif) or rabbit IgG isotype control (# 3900, Cell Signaling Technology).

ChiP DNA was analyzed by real-time PCR using GoTaq^®^ qPCR Master Mix (Promega). The primers used for DNA amplification are listed in Supplementary Table S3. QPCR reactions were run using LightCycler 480 (Roche Applied Sciences) following the amplification program: 10 min at 95°C, followed by 50 cycles at 95°C for 20 sec and 60°C for 1 min. Results were normalized against a standard curve generated using dilution of the input for each primer and represented as % input.

### RNA-seq analysis

LNCaP cells were transduced with lentivirus expressing AR-WT and AR-V7 in complete medium containing 10 nM DHT. Three days after transduction, total RNA was extracted using TriPure Reagent (Roche) according to the manufacturer's protocol. For RNA-seq analysis, three samples per condition were analyzed. The library of cDNA and the sequencing were performed by IGBMC Microarray and Sequencing platform (Illkirch, France).

RNA-seq reads were mapped onto the hg19 assembly of the human genome using Tophat v2.0.10 [50] and the bowtie2 v2.1.0 aligner [51]. Quantification of gene expression was performed using HTSeq v0.5.4p3 [52] using gene annotations from Ensemble release 75. The normalization of read counts across libraries was performed with the method proposed by Anders and Huber [53]. For statistical analysis, comparison of samples was performed using the method proposed by Love et al. [54] implemented in the DESeq2 Bioconductor library (DESeq2 v1.0.19). Resulting *p*-values were adjusted for multiple testing by using the Benjamini and Hochberg (1995) method [55] (GEO dataset GSE71334). Functional and pathway analysis of differentially expressed genes in the presence of AR variants were performed using Ingenuity^®^ Pathway Analysis tool (IPA, www.ingenuity.com, Qiagen).

### miRNA-seq analysis

RNA samples used for RNA-seq were also analyzed by miRNA-seq. As mentioned above, the library of cDNA and the sequencing were performed by IGBMC Microarray and Sequencing platform.

For data analysis, adapters were trimmed from total reads using FASTX_Toolkit (http://hannonlab.cshl.edu/fastx_toolkit/). Only trimmed reads with a length between 15 and 40 nucleotides were kept for the further analysis. Data analysis was performed according to published pipeline ncPRO-seq [56]. Briefly, reads were mapped onto the Hg19 genome assembly with Bowtie v0.12.8 [57]. The annotations were done with miRBase release 20 for the microRNAs, with Repbase for the repeats and with Rfam for the other small non-coding RNAs. The normalization and differential expression analysis were done with DESeq2 R package v1.0.12 [53] (GEO dataset GSE71335).

### Integrative analysis

Integrative analysis between RNA-seq and miRNA-seq was performed by comparing a list of differentially expressed genes (DEG) and differentially expressed miRNA (DEM). The list of DEG comprised all the genes with a fold change ≤ or ≥ 1.5 and adjusted *p*-value ≤ 0.05 and for the list of DEM, we have selected all the miRNA differentially expressed with adjusted *p*-value ≤ 0.05. These two lists were separately uploaded in Ingenuity Pathway Analysis (IPA) software (www.ingenuity.com, Qiagen) and the putative targets of DEM were identified using microRNA Target Filter. Briefly, this tool is the combination of four databases based on prediction binding (TargetScan) and experimentally validated interaction (miRecords, Tarbase and Ingenuity^®^ Knowledge base). For further analyses, only pairs of mRNA-miRNA with anticorrelated expression were selected.

## SUPPLEMENTARY TABLES AND FIGURES



## Abbreviations

AR: Androgen Receptor
AR-FL: full-length Androgen Receptor
ARE: Androgen Response Element
CRPC: Castration Resistant Prostate Cancer
EMT: Epithelial Mesenchymal Transition
PCa: Prostate Cancer
