# Supplementary material for: Dual effects of constitutively active androgen receptor and full-length androgen receptor for N-cadherin regulation in prostate cancer
Source: Oncotarget. 2017 May 29;8(42):72008–20. doi: 10.18632/oncotarget.18270 (PMC5641107; doi:10.18632/oncotarget.18270)
Supplement: Supplementary file 1 [file oncotarget-08-72008-s001.pdf]

Dual effects of constitutively active androgen receptor and full-length androgen receptor for N-cadherin regulation in prostate cancer

Supplementary Materials

MATERIALS AND METHODS

Plasmids

To study the importance of DNA binding properties of AR variants for upregulation of N-cadherin, cysteine in position 576 was substituted to a tyrosine to yield to the AR-V7-C576Y mutant. This mutant was unable to bind DNA. Briefly, the mutation was introduced in AR-V7 sequence using the following polymerase chain reaction (PCR) primers: a HindIII site-linked primer containing the G2845A mutation 5′GCCTGATCTGTGGAGATGAAGCTTCTGGGTGTC ACTATGGAGCTCTCACAT ATGGAAGCTCAA3′ (NM_000044.3; nucleotides 2784-2853) as the forward primer and a BamHI site-linked primer 5′CAGTTATCTAGATCCGGTGGATC*CCTTTCTTCAG GGTCTGGTCATTTTGAGATGCTTGCAATTGC* 3′ (FJ235916.1; nucleotides 2230-2272 in italics; U57607.1; nucleotides 1387-1409 underlined) as the reverse primer. pE-AR-V7-C576Y and pEGFP-AR-V7-C576Y were constructed from previously described pE-AR-V7 and pEGFP-AR-V7 respectively, by replacing the HindIII/BamHI fragment with` the C576Y mutant sequence using In fusion® HD Cloning Kit (Clontech).

For luciferase assay, the *CDH2* promoter (NC_000018.10, position 28177782-28177023) and intron 1 *CDH2* genomic sequence (NC_000018.10, position 28157248-28156350) containing 13 AREs were inserted into pGL4.14 vector (Promega) to yield to the pARE_CDH2luc plasmid. *CDH2* promoter and intron 1_*CDH2*_ARE sequences were amplified from genomic DNA of LNCaP cells using the following primers: the forward primer incorporating KpnI restriction site 5′TATTATGGTACCACGGAGCCGCGGCGGGAG AGACCGC3′ and the reverse primer incorporating Hind III restriction site 5′TATTATAAGCTTGGAGG CGGAG AGGGGCCGAGCGAAGA3′ for promoter; the forward primer with BamHI restriction site 5′TATTATGGATCCTATAAGCGTGTTGCAGTCTCTG CTG3′ and the reverse primer with SalI restriction site 5′TATTACGTCGACGGACAAAAATCTGGGTCAAAA TCTGGG3′ for ARE sequence. The amplicons were cloned by ligation into pGL4.14 vector between KpnI/HindIII and BamHI/SalI for *CDH2* promoter and ARE sequence respectively.

Transient transfection

LNCaP cells were transfected using JetPEI™ (Polyplus Transfection, Ozyme) according to the manufacturer's protocol. Briefly, $2.5x10^5$ LNCaP cells were seeded in 6-wells plates and transfected three days later with 3 µg of pE-AR-V7 or pE-AR-V7-C576Y plasmid using 6 µL of JET-PEI (Polyplus Transfection, Ozyme). Cells were incubated during 4 days in complete medium supplemented with 10 nM dihydrotestosterone (DHT) and RNA was isolated using NucleoSpin® RNA II assay (Macherey-Nagel).

Luciferase assay

LNCaP cells (1.5×10^4/well) were plated in 96-wells plates for three days. Cells were then co-transfected with 75 ng of pE-AR-V7 or pE-AR-V7-C576Y, 150 ng of luciferase reporter plasmid pARE_CDH2luc and 7.5 ng of Renilla luciferase plasmid as an internal control using 0.5 µL of JET-PEI/well. Twenty-four hours after transfection, medium was replaced with phenol red-free RPMI 1640 containing 5% charcoal-stripped FBS (CSS) with 10 nM DHT. Firefly and Renilla luciferase activities were measured 72 h after transfection using Dual-Glo Luciferase Assay according to the manufacturer's protocol (Promega).

Supplementary Table 1: Sequence of primers used for cloning of pLVX-TRE3G-AR

Name	Sequence	Position Information
FOR-LVXTRE3G	5′ *TCTTATACTTGGATCC***ATGGTGA GCAAGGGCGAGGAGCT** 3′	pLVX-TRE3G: nucleotides 2576-2561 in italics; EGFP sequence in bold characters
REV-LVX_WT	5′ *ATTCCATATGACGCGT***TCACTGG GTGTGGAAATAGA** 3′	pLVX-TRE3G: nucleotides 2582-2597 in italics; NM_000044.3: nucleotides 3859-3879 in bold characters
REV-LVX_Q640X	5′ *ATTCCATATGACGCGT***CTATAG TTTCAGATTACCA** 3′	pLVX-TRE3G: nucleotides 2582-2597 in italics; NM_000044.3: nucleotides 3020-3038 in bold characters
REV-LVX_V7	5′ *ATTCCATATGACGCGT***TCAGGG TCTGGTCATTTTGA** 3′	pLVX-TRE3G: nucleotides 2582-2597 in italics; FJ235916.1: nucleotides 2246-2266 in bold characters

BamHI (lane 1) and MluI (lanes 2,3,4) restriction sites were underlined.

Supplementary Table 2: List of primers used for qRT-PCR

Name	Genbank or *Sanger Accession*	GeneGlobe Reference/*Sequence*	Position or *Exons*	Amplicon length (bp)
β-ACTIN (ACTB)	NM_001101	QT01680476	NA	104
N-CADHERIN (CDH2)	NM_001792	QT00063196	*14/15*	102
ETV5	NM_004454.2	QT0009485	*7/8*	89
GFP	NA	QT01171611	NA	NA
PBGD (HMBS)	NM_000190	QT00014462	*7/8/9*	107

Supplementary Table 3: Sequence of primers used for ChiP experiment

Name	Sequence	Accession number	Position	Amplicon length (bp)
ARE of *CDH2* forward	5′ AGACCCAGATTTTGACCCAG 3′	NC_000018.10	28156379–28156360	97pb
ARE of *CDH2* reverse	5′ CAGGAACCTGCAAACTGTTGA 3′	NC_000018.10	28156282–28156302	
Promoter of *β-GLOBIN* forward	5′ AGGACAGGTACGGCTGTCATC 3′	NC_000011.10	5227207–5227187	90pb
Promoter of *β-GLOBIN* reverse	5′TTTATGCCCAGCCCTGGCTC 3′	NC_000011.10	52277098–5227117	

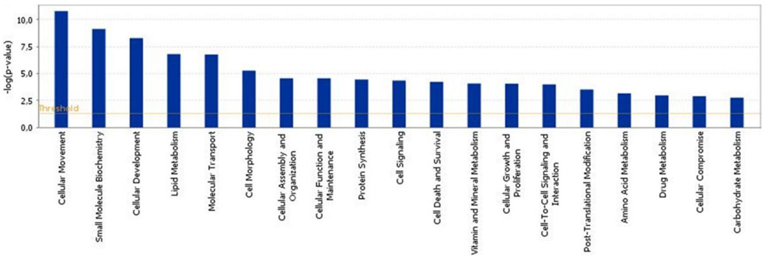

Supplementary Figure 1: Functional analysis of deregulated genes in the presence of AR-V7. RNA-seq analysis was performed in LNCaP cells overexpressing AR-WT and AR-V7. From this experiment, we identified 751 upregulated genes and 108 downregulated genes in cells overexpressing AR-V7 compared to AR-WT (log2Fold-Change >1 and adjusted p-value for multiple testing $P < 0.05$). The list of deregulated genes was uploaded in IPA software to check altered functions in the presence of AR-V7. Interestingly, the expression of AR-V7 affects functions involved in cellular movement or cell morphology.

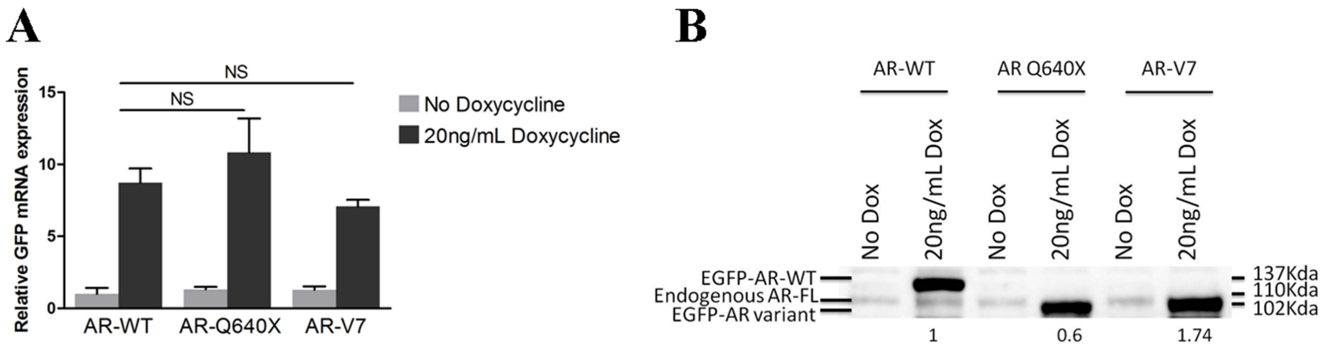

Supplementary Figure 2: Expression of AR-WT, AR-Q640X and AR-V7 using lentiviral inducible system. The Lenti-X™ Tet-ON® 3G Inducible Expression System was used to generate a tetracycline-regulated AR expression system in the LNCaP cells. AR-WT, AR-Q640X and AR-V7 expression were induced with 20 ng/mL of doxycycline. (**A**) After 4 days of induction by doxycycline, EGFP RNA level was analyzed by qRT-PCR. (**B**) A Western Blot using TGX Stain-Free™ FastCast™ Acrylamide Gel was performed to assess AR protein levels. Total protein normalization (indicated at the bottom of each lane) was quantified according the manufacturer's protocol and reported as a relative value to doxycycline induced AR-WT. Western Blot image was cropped to eliminate the parts containing no information. NS: Not statistically significant.

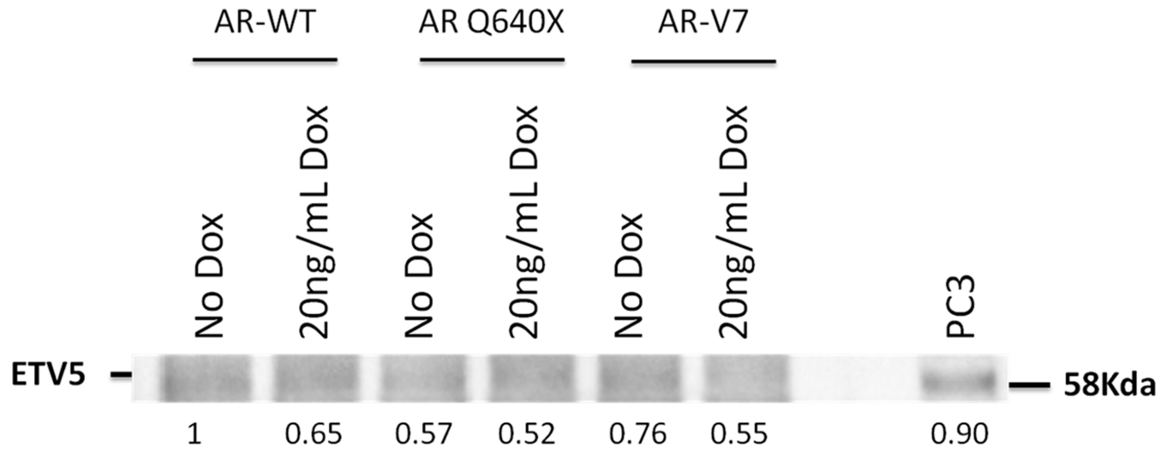

Supplementary Figure 3: ETV5 expression in the presence of AR-WT or AR variants. AR-WT and AR variants were induced with 20 ng/mL of doxycycline and proteins were extracted after 4 days of induction. A Western Blot (cropped image) using TGX Stain-Free™ FastCast™ Acrylamide Gel was performed to assess ETV5 protein expression level. Prostate cancer cells PC3 were used as positive control for ETV5 expression.

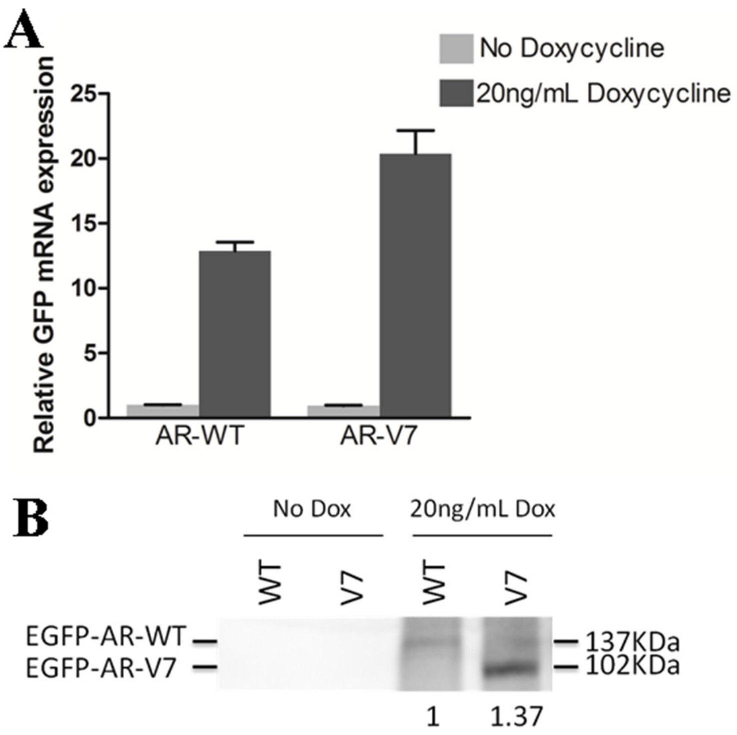

Supplementary Figure 4: AR-WT and AR-V7 expression after doxycycline induction. EGFP-AR-WT and EGFP-AR-V7 were induced using 20 ng/mL of doxycycline. Twenty-four hours after induction, GFP expression was analyzed by qRT-PCR (**A**) or by Western Blot from chromatin extracts (**B**). A band at 137 KDa and 102 KDa was respectively detected in lane 3 (AR-WT) and lane 4 (AR variants) attesting the absence of degradation of AR. No band was detected in non-induced LNCaP cells used as negative control. For Western Blot, TGX Stain-Free™ FastCast™ Acrylamide gel was used and total protein normalization (indicated at the bottom of each lane) was performed according the manufacturer's protocol. Western Blot image was cropped to only show the region containing AR bands.

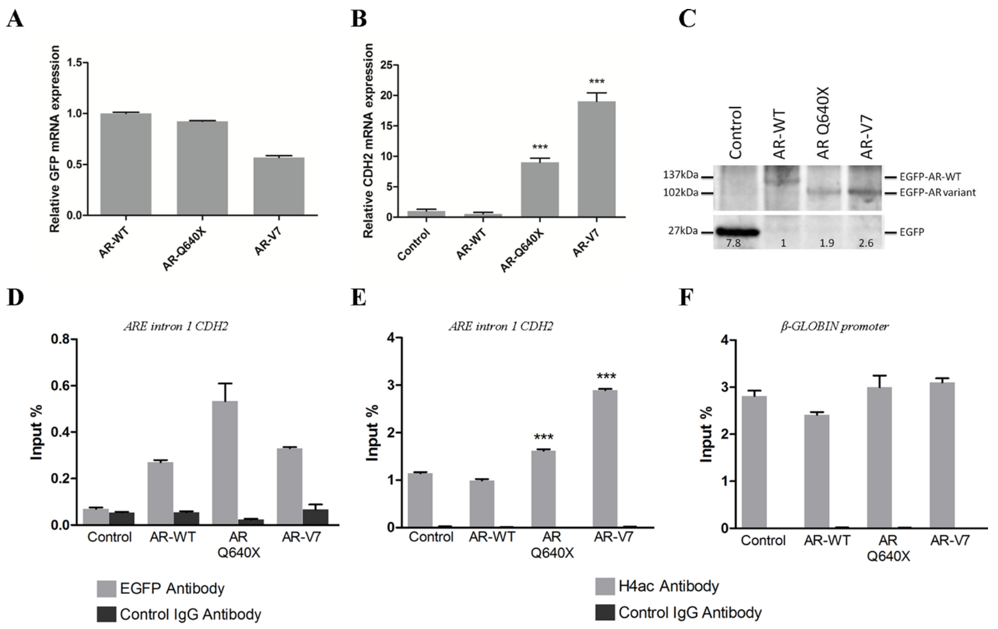

Supplementary Figure 5: AR variants bind to ARE in intron 1 of *CDH2* and are associated with increased histone H4 acetylation in LNCaP cells. LNCaP cells were transduced with lentivirus expressing AR-WT, AR variants (AR-Q640X; AR-V7) or EGFP (Control). Transduced cells were cultured in complete medium supplemented with 10 nM DHT. After 72 h, qRT-PCR was performed to verify the expression level of GFP (**A**) and CDH2 (**B**). The ΔΔCt method was used to calculate relative expression and the results were presented as the mean of ΔΔCt ± SEM. *** $P < 0.001$, two-tailed Student's *t* -test. (**C**) Western Blot analysis from chromatin extracts using EGFP antibody. Intensity of each band was normalized to total proteins using TGX Stain-Free™ FastCast™ Acrylamide gel and indicated at the bottom of each lane. Image was cropped to only show the part of the blot containing AR bands. (**D**) Binding of AR-WT and AR variants to AREs in *CDH2* intron 1 was assessed by ChiP experiment using anti-EGFP antibody and analyzed by real-time PCR. Acetylation level at AREs in *CDH2* intron 1 (**E**) and *β-GLOBIN* promoter (**F**) was assessed by ChiP experiment using anti-Acetyl H4 (H4ac) antibody and examined by qPCR with specific primers. EGFP and histone H4 acetylation level at AREs in *CDH2* intron 1 and β-GLOBIN promoter were normalized by input. *** $P < 0.001$.

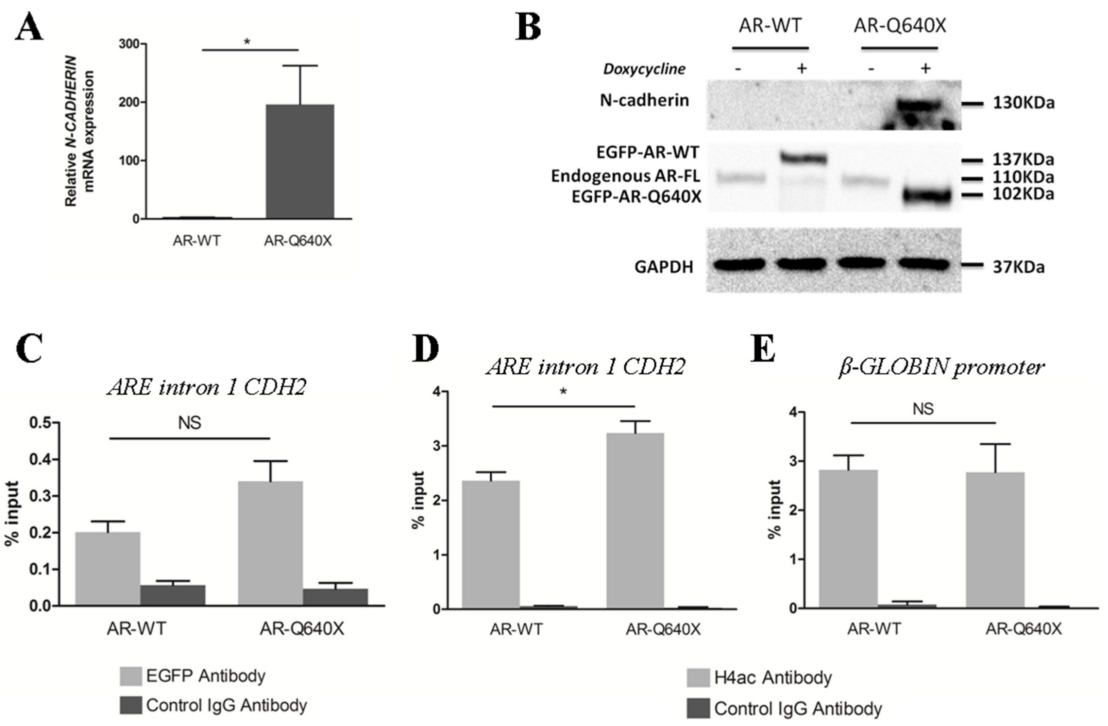

Supplementary Figure 6: AR-Q640X variant binds to ARE in intron 1 of *CDH2* and leads to an increased histone H4 acetylation level in C4-2B cells. C4-2B cells were transduced with inducible EGFP-AR-WT and EGFP-AR-Q640X lentiviral particles. The expression of AR-WT and AR-Q640X was induced with 20 ng/mL of doxycycline and cells were cultured in the presence of 10 nM DHT. mRNA and protein extractions were performed 72 h after induction to analyze N-cadherin and AR expression by qRT-PCR (**A**) and Western Blot (cropped image) (**B**). (**C**) ChiP-qPCR experiment was conducted with anti-EGFP antibody to analyze AR-WT and AR-Q640X recruitment at ARE in *CDH2* intron 1. (**D**) H4 acetylation level in C4-2B cells expressing AR-WT or AR-Q640X was analyzed at ARE in *CDH2* intron 1 using anti-Acetyl H4 (H4ac) antibody. (**E**) H4 acetylation level was determined at *β-GLOBIN* promoter as control. *$P < 0.05$, NS: Not statistically significant.

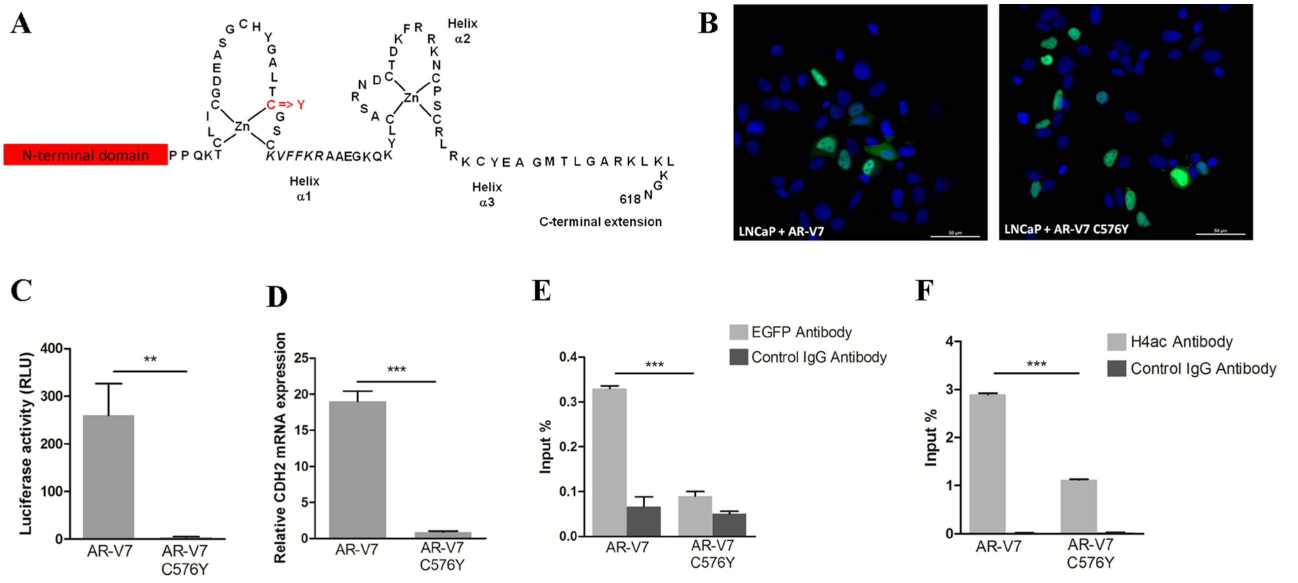

Supplementary Figure 7: N-cadherin upregulation requires AR functional DNA binding domain. (**A**) Schematic representation of AR-V7 C576Y. This variant contains a mutation in position 576 (cysteine → tyrosine) in DNA binding domain. (**B**) Subcellular location of EGFP-AR-V7 or EGFP-AR-V7 C576Y was assessed by fluorescence microscopy 24 h after transient transfection of LNCaP cells. AR-V7 and AR-V7 C576Y were both located in the nucleus, suggesting that translocation properties of AR variants were not impaired in the presence of C576Y mutation. (**C**) LNCaP cells were co-transfected with pE-AR-V7 or pE-AR-V7 C576Y and pARE_CDH2luc reporter and cultured in phenol red-free RPMI 1640 containing 5% charcoal-stripped FBS (CSS) with 10 nM DHT. Luciferase activities were measured 72 h after transfection. The luciferase activity was normalized to the Renilla luciferase activity, and the results are presented as means ± SEM. Contrary to AR-V7, AR-V7 C576Y was unable to induce a luciferase activity. $^{**}P < 0.01$. (**D**) N-cadherin (*CDH2*) expression was analyzed by qRT-PCR in LNCaP cells expressing AR-V7 or AR-V7 C576Y. *CDH2* mRNA expression level was normalized to *PBGD*. Relative expression is presented as the mean ± SEM from three independent experiments. (**E**, **F**) LNCaP cells were transduced with lentivirus expressing AR-V7 or AR-V7 C576Y and cultured in complete medium supplemented with 10 nM DHT. After 72 h hours, AR recruitment at AREs (E) and histone H4 acetylation level (F) were assessed by ChiP-qPCR. $^{***}P < 0.001$.

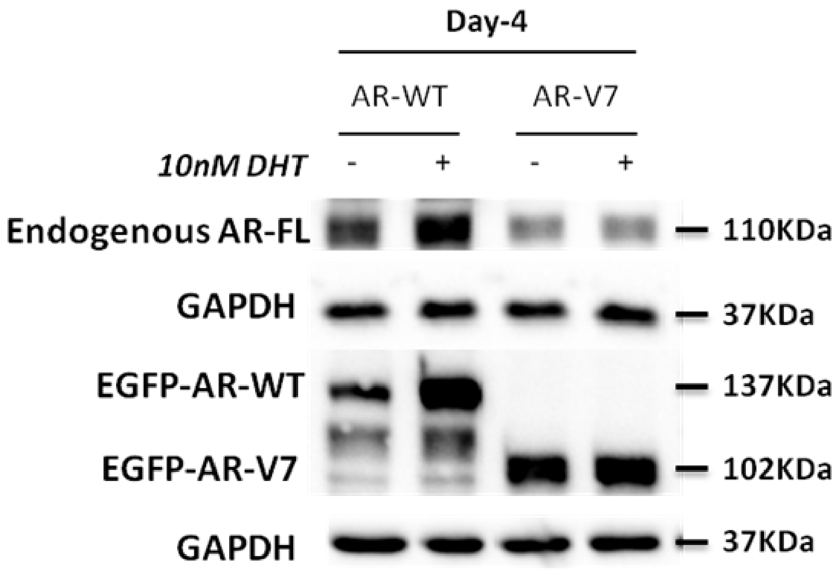

Supplementary Figure 8: Downregulation of AR-FL in the presence of AR variants. EGFP tagged AR-WT and AR-V7 expression were induced with 20 ng/mL doxycycline and cells were maintained in medium containing 5% charcoal stripped serum supplemented with 10 nM of DHT or EtOH as control during 4 days. Endogenous AR-FL and transgene expression was analyzed by Western Blot using respectively a specific antibody targeting the C-terminal extremity of AR (AR-C19 antibody) or EGFP. GAPDH was used as a loading control. Western Blot image was cropped to only show the regions of interest.
